# White Matter Ischemic Changes in Hyperacute Ischemic Stroke: Voxel-Based Analysis Using Diffusion Tensor Imaging and MR Perfusion

**DOI:** 10.1161/STROKEAHA.114.007000

**Published:** 2015-01-26

**Authors:** Kambiz Nael, Theodore P Trouard, Scott R. Lafleur, Elizabeth A. Krupinski, Noriko Salamon, Chelsea S. Kidwell

**Affiliations:** From the Departments of Medical Imaging (K.N., T.P.T., E.A.K., C.S.K.), Biomedical Engineering (T.P.T., S.R.L., E.A.K.), Neurology (C.S.K.), University of Arizona, Tucson; and Department of Radiology, University of California, Los Angeles (N.S.).

**Keywords:** diffusion imaging, ischemia, magnetic resonance imaging, perfusion imaging, stroke

## Abstract

**Background and Purpose—:**

The purpose of this study was to evaluate changes in fractional anisotropy (FA), as measured by diffusion tensor imaging, of white matter (WM) infarction and hypoperfusion in patients with acute ischemic stroke using a quantitative voxel-based analysis.

**Methods—:**

In this prospective study, diffusion tensor imaging and dynamic susceptibility contrast perfusion sequences were acquired in 21 patients with acute ischemic stroke who presented within 6 hours of symptom onset. The coregistered FA, apparent diffusion coefficient, and dynamic susceptibility contrast time to maximum (Tmax) maps were used for voxel-based quantification using a region of interest approach in the ipsilateral affected side and in the homologous contralateral WM. The regions of WM infarction versus hypoperfusion were segmented using a threshold method. Data were analyzed by regression and ANOVA.

**Results—:**

There was an overall significant mean difference (*P*<0.001) for the apparent diffusion coefficient, Tmax, and FA values between the normal, hypoperfused, and infarcted WM. The mean±SD of FA was significantly higher (*P*<0.001) in hypoperfused WM (0.397±0.019) and lower (*P*<0.001) in infarcted WM (0.313±0.037) when compared with normal WM (0.360±0.020). Regression tree analysis of hypoperfused WM showed the largest mean FA difference at Tmax above versus below 5.4 s with a mean difference of 0.033 (*P*=0.0096).

**Conclusions—:**

Diffusion tensor imaging-FA was decreased in regions of WM infarction and increased in hypoperfused WM in patients with hyperacute acute ischemic stroke. The significantly increased FA values in the hypoperfused WM with Tmax≥5.4 s are suggestive of early ischemic microstructural changes.

Diffusion-weighted imaging measures the restriction of water movement associated with cytotoxic edema and has become the most sensitive imaging technique for detection of early cerebral ischemia.^1^ Regions of restricted diffusion on diffusion-weighted imaging are associated with a decrease in Na-K-ATPase activity and energy failure, and in most cases represent irreversible infarction.^2^ Although several studies have demonstrated that apparent diffusion coefficient (ADC) values of <600×10^−6^ mm^2^/s typically represent irreversible infarction^3^; the ischemic changes remain rather ill-defined and the associated pathophysiology is not well understood.

MR perfusion, in particular dynamic contrast susceptibility (DSC) perfusion, has been useful in evaluation of cerebral hemodynamic changes after acute ischemic stroke (AIS)^4,5^ and defining the operational ischemic penumbra as regions characterized by normal diffusion but abnormal perfusion.^6,7^ This concept of perfusion–diffusion mismatch, however, remains controversial^8,9^ with lack of consensus on what perfusion parameters should be used to define the penumbra and to distinguish the penumbra from regions of benign oligemia (tissue with decreased perfusion but not at risk of infarction). Using parametric time maps, such as time-to-peak of the residue function (Tmax), a wide range of Tmax values (2–8 s) has been proposed to define the threshold for identifying the potential ischemic penumbra.^10–12^

Fractional anisotropy (FA) obtained from diffusion tensor imaging (DTI) may provide an additional means of defining and understanding the microstructural changes in ischemic brain acutely, particularly in white matter (WM).^13^ The FA measures the degree of directionality of diffusion and, therefore, WM tract integrity. In normal tissue, the WM FA is high, whereas it becomes reduced with loss of integrity or disorganization of tracts. However, FA changes are heterogeneous and variable between the infarction core and ischemic regions depending on the severity of ischemia and time of onset.^14,15^ Thus, additional study is required to delineate the use of these techniques in AIS.

The purpose of this study, therefore, was to perform a voxel-based quantitative analysis of combined use of DTI-FA and DSC-Tmax in WM infarction and hypoperfusion in patients with AIS to test the following hypotheses: (1) FA values are spatially different between the infarction core versus hypoperfusion versus normal WM; and (2) FA can identify microstructural changes associated with hypoperfused ischemic, but not yet infarcted, WM. To demonstrate this, we aimed to find a Tmax threshold at which the highest differences in FA could be identified.

## Methods

### Patients

This prospective study was conducted between December 2012 and July 2013. Patients with suspected AIS were enrolled. Inclusion criteria were (1) interval between the onset of neurological deficits to MRI of <6 hours; (2) image acquisition at 3.0T magnetic field with both DSC and DTI studies obtained; and (3) presence of infarction and perfusion–diffusion mismatch as identified by MRI. Patient demographic data, median time from last known well to first MRI, and baseline National Institutes of Health Stroke Scale scores were documented.

### Imaging Protocol

All patients underwent MRI on a 3.0T Siemens Skyra MRI system (Siemens, Erlangen, Germany). The imaging protocol included DTI, fluid attenuation inversion recovery imaging, gradient recalled echo, MR angiography, and DSC perfusion imaging.

DTI was acquired using single-shot spin-echo echo-planar imaging (repetition time/echo time, 5500/82 ms; field of view, 22×22 cm; matrix, 128 mm; slices, 40×3 mm; voxel size, 1.5×1.5×3 mm). Diffusion gradients were applied along 20 noncollinear directions with a *b* value of 1000 s/mm^2^ resulting in a 5-minute acquisition time. A generalized partial parallel acquisition^16^ technique with acceleration factor of 3 was used.

DSC perfusion was performed using a single-shot gradient-echo echo-planar imaging sequence with the following parameters: repetition time/echo time, 1450/22 ms, FA=90°; field of view, 22×22 cm; matrix, 128×128 mm, 30×4 mm slices; generalized partial parallel acquisition, 3). A total of 60 repetitions were acquired after intravenous injection of 0.1 mmol/kg of gadolinium contrast agent at a rate of 5 mL/s.

### Data Analysis

DSC and DTI studies were processed using commercially available Food and Drug Administration–approved software (Olea Sphere; Olea Medical SAS, La Ciotat, France). DSC analysis was performed using a block-circulant singular value decomposition technique.^17^ The Tmax maps were then automatically generated and exported from the software for subsequent analysis. DTI analysis was also performed by the Olea DTI package, where FA and ADC maps were calculated using standard methods.^18^

**Figure 1.**
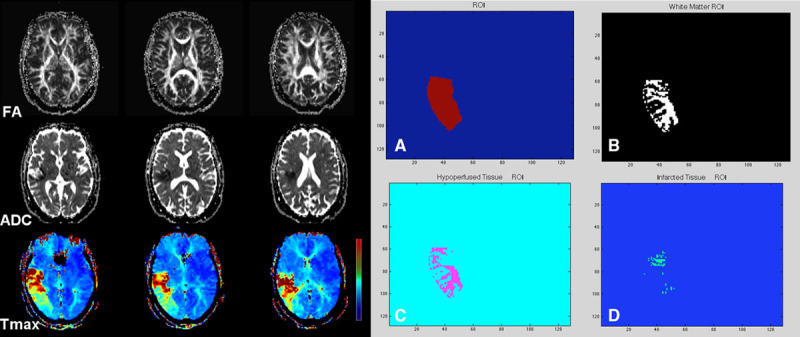
A 71-year-old woman with sudden onset left-sided weakness, baseline National Institutes of Health Stroke Scale, 17. Concurrent MRA showed left M1 occlusion (not shown). Sequential aligned diffusion tensor imaging-fractional anisotropy (FA), apparent diffusion coefficient (ADC), and dynamic contrast susceptibility (DSC)-Tmax are shown. There is acute infarction in the right corona radiata and subinsular region with a large hypoperfusion deficit along the right middle cerebral artery territory on DSC-Tmax images. Aligned and coregistered maps were transferred to a Matlab program. Using DSC-Tmax, a regions of interest (ROI) was drawn over the hypoperfused region (**A**). After subtraction of a gray matter mask, an ROI subsuming the white matter voxels was generated (**B**). The FA and Tmax values were then calculated in the region of hypoperfusion (**C**) and infarction (**D**) after inclusion of an ADC map with threshold of greater and <600×10^−6^ mm^2^/s, respectively.

FA, ADC, and Tmax maps for each patient were coregistered with the Olea software using a 12 degree of freedom transformation and a mutual information cost function. This was followed by visual inspection to ensure adequate alignment. Coregistered images were exported into Matlab program for voxel-based quantitative analysis. An example of image analysis segmentation of WM infarction versus hypoperfusion is shown in Figure 1. A mask of the gray matter (FA threshold >0.15) was generated for each patient to ensure extraction of voxel values was limited only to WM. A map of the infarction core was also generated by a threshold method defined as an ADC value <600×10^−6^ mm^2^/s.^3^ For WM infarction, the quantitative values were calculated after applying the gray matter mask (FA>0.15) and within the regions with ADC<600×10^−6^ mm^2^/s. For the WM hypoperfusion, the quantitative values were calculated after applying the gray matter mask (FA>0.15) and within the regions with ADC>600×10^−6^ mm^2^/s. Regions of interest were placed over the area of perfusion abnormality using the coregistered Tmax maps and also in contralateral normal WM in the centrum semiovale. Perfusion deficit was defined as an area with visually perceptible increased Tmax when compared to the surrounding brain tissue and to the homologous contralateral hemisphere. For each regions of interest, the FA, ADC, and Tmax values in the regions of infarction, hypoperfusion, and normal WM were calculated and exported into an excel spreadsheet for statistical analysis.

### Statistical Analysis

Mean values of ADC, FA and Tmax were first computed across voxels for each person and region. Then these person–region means were used in a mixed (repeated measure) ANOVA model. Examination of residual errors under this model confirmed that the residual errors had a normal distribution, justifying the use of a parametric model. Multiple comparison Tukey-adjusted *P* values were reported. The significance level was defined as *P*<0.05. Spearman correlations (*r*_s_) were computed, and scatter plots were examined to assess the association between ADC, FA, and Tmax in normal, hypoperfused, and infarcted WM. A regression tree analysis was used to find the value of Tmax that best splits FA into high and low values.

## Results

Twenty-one patients (14M, 7F) with a mean age of 62.4 (range 47–83) years met our inclusion criteria. The baseline National Institutes of Health Stroke Scale scores ranged from 4 to 17 with a median of 7. The median time from last well known to first MRI was 4.7 hours (range 1–6 hours). The median volume of infarction based on the threshold ADC<600×10^−6^ mm^2^/s was 19.2 mL (range, 11–58 mL).

The mean±SD of the ADC, FA, and Tmax values for the normal, hypoperfused, and infarcted WM are shown in Table 1. Repeated measure ANOVA model revealed statistically significant differences between mean values of ADC, FA, and Tmax across all regions except for the ADC difference between normal versus hypoperfused WM (*P*=0.65; Table 2).

**Table 1.**
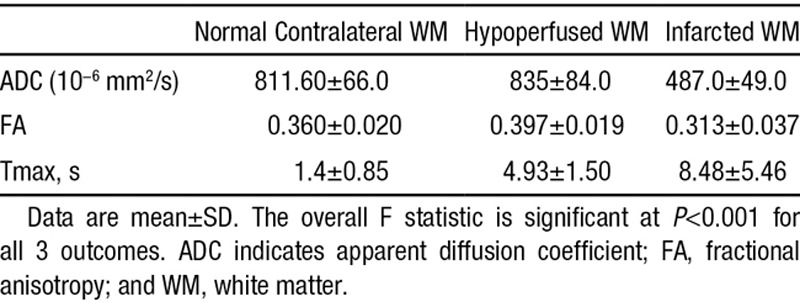
Mean±SD of ADC, FA, and Tmax in Normal, Hypoperfused, and Infarcted WM

**Table 2.**
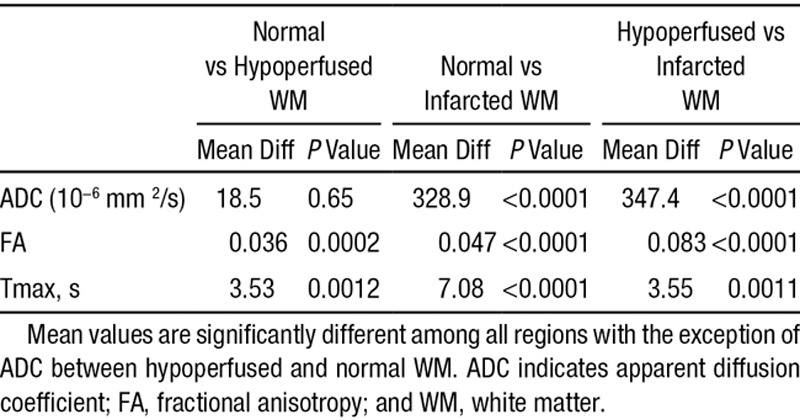
Mean Differences for Paired-Wise Comparison Between ADC, FA, and Tmax Across Different WM Regions

**Figure 2.**
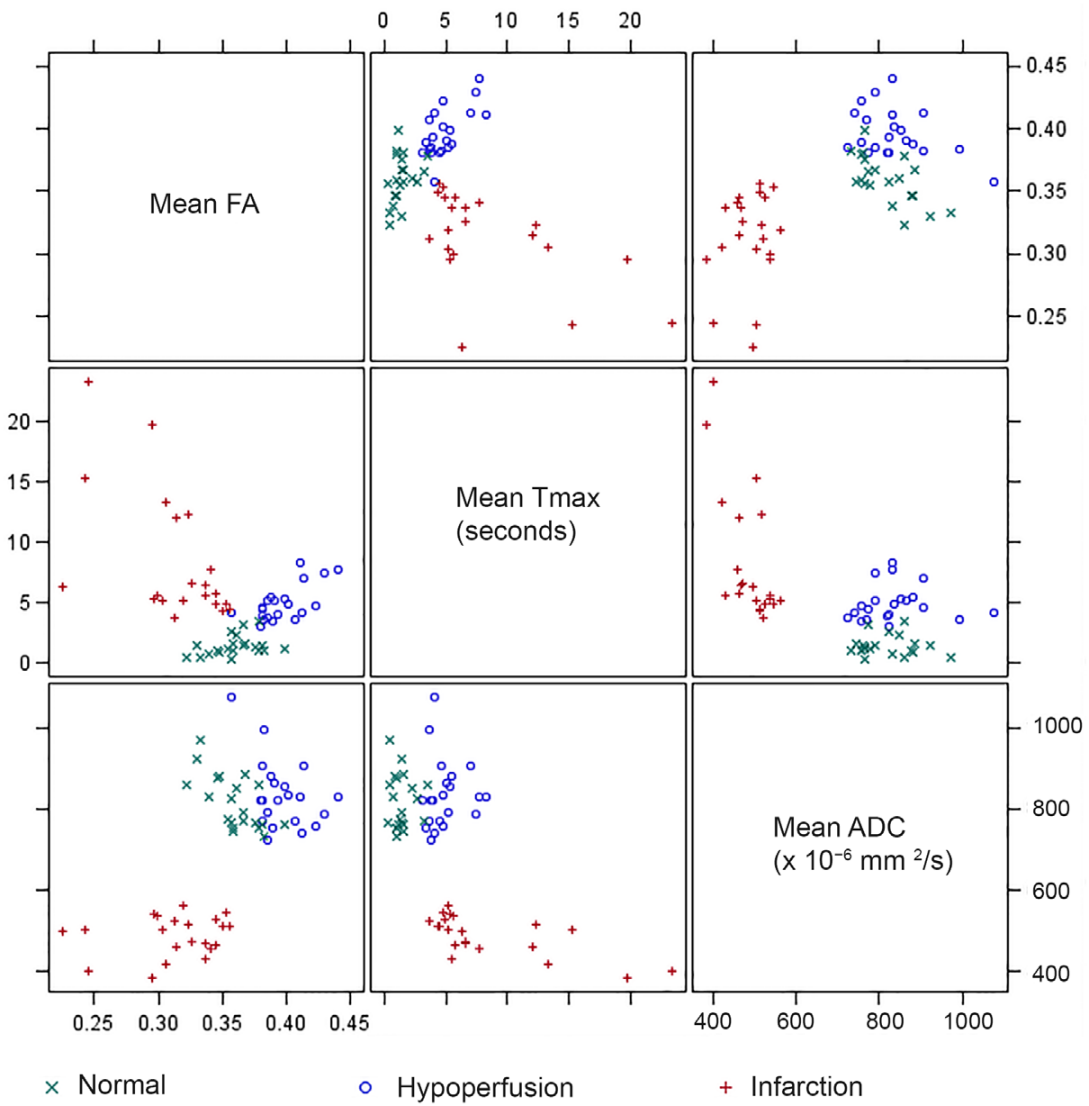
Scatter plots for fractional anisotropy (FA), Tmax, and apparent diffusion coefficient (ADC) mean values by region. The correlation pattern is different depending on the region. In the normal white matter (WM), FA, and ADC have a moderate negative correlation (*r*_s_=−0.590; *P*=0.005). In the hypoperfused WM, Tmax and FA have moderate positive correlation (*r*_s_=0.561; *P*=0.008). In the infarcted WM, there are moderate negative correlations between Tmax and FA (*r*_s_=−0.539; *P*=0.012) and Tmax and ADC (*r*_s_=−0.681; *P*<0.001).

Scatter plots for association between ADC, FA, and Tmax in normal, hypoperfused, and infarcted WM are shown in Figure 2. Spearman correlations (*r*_s_) for association between ADC-FA, ADC-Tmax, and FA-Tmax were −0.590, −0.021, and 0.418 in normal WM; −0.230, 0.319, and 0.561 in hypoperfused WM, and 0.209, −0.681, and −0.539 in infarcted WM, respectively.

**Figure 3.**
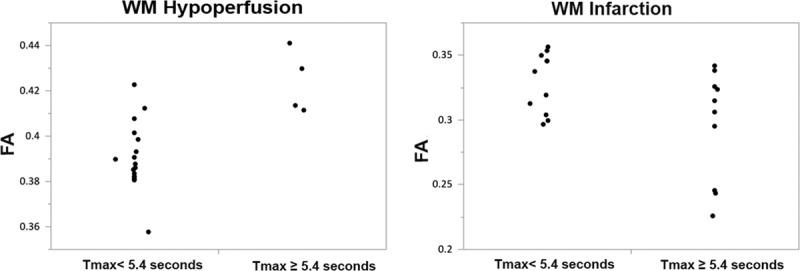
Mean fractional anisotropy (FA) value comparisons in hypoperfused and infarcted white matter (WM) for Tmax<5.4 vs Tmax≥5.4 s. The mean±SD for FA values in hypoperfused WM is 0.390±0.014 for Tmax<5.4 s when compared with 0.423±0.014 in Tmax≥5.4 (*P*=0.0096). In the infarcted WM, the mean±SD for FA values are 0.328±0.023 for Tmax<5.4 s compared with 0.295±0.042 in Tmax≥5.4 (*P*=0.023).

In a subanalysis of hypoperfused WM using regression tree analysis, the largest mean difference in FA values was identified at Tmax above and below 5.4 s (*P*=0.0096), the Tmax threshold that best split FA such that the residual variance was minimized. Figure 3 shows the mean FA value comparisons in hypoperfused and infarcted WM for Tmax<5.4 s versus Tmax≥5.4 s.

## Discussion

Advanced imaging techniques, such as DSC perfusion and DTI, can be used to interrogate the spatial heterogeneity of infarction and ischemia in the setting of hyperacute ischemic stroke further. In this study, using a combination of DTI and DSC perfusion, we conducted a voxel-based analysis of DTI-measured FA and DSC-measured Tmax changes in the regions of WM infarction and hypoperfusion using defined thresholds. In addition, we compared FA and Tmax to identify a time-based threshold for the detection of microstructural changes. We note 2 primary findings.

The first is that FA values in the hypoperfused and infarcted WM are significantly different from normal WM, but in opposite directions. Reduced FA values in infarcted WM likely signify the loss of cellular integrity with irreversible cellular injury.^13^ On the contrary, we showed that FA values are significantly elevated in the hypoperfused (but not infarcted) WM in comparison with normal WM. This is in agreement with previous studies of animal models of brain ischemia.^14^ The elevation in FA occurs in the context of a reduction in the anisotropic tensor and therefore is a consequence of ratio-metric measurement.^19^ The acute increase in FA has been linked to cytotoxic edema without a significant change in structural coherence.^20^ Subsequent to cytotoxic edema, there could be an increase in tortuosity of the extracellular space and shift of water from the extracellular space to the more restricted intracellular space. Both of these models result in increased directionality of diffusion along the axon and hence increased apparent anisotropy.^20,21^

Our second finding is that the FA values are significantly higher in the hypoperfused WM with Tmax≥5.4 s in comparison with regions with Tmax<5.4 s with a mean difference of 0.033. Parametric MR perfusion maps, such as Tmax, have commonly been used to identify the penumbral tissues in some clinical trials.^22–24^ One major drawback of using time-domain perfusion parameters, such as Tmax, is the fact that a perfusion deficit may represent any part of hemodynamic milieu from delayed perfusion to benign oligemia to hypoperfusion and likely a combination of all of the above. Thus, not all regions with Tmax delay are necessarily destined for infarction. Tmax solely provides an estimate of the delay in bolus arrival time between the arterial input function and a given voxel, without describing the hemodynamic status of the tissue or degree of ischemia itself.^25^ Many investigators have attempted to identify a predefined threshold for Tmax that represents true ischemic penumbra, and as the result, Tmax values with threshold ranging from 2 to 8 s have been used in the literature.^10–12^ Most of these investigations were focused on the correlation with final infarction core size at day 7 or 30 after the ischemic event.

There has been relatively little attention on the role of combined DTI and MR perfusion to characterize ischemic tissue. In this study, we prospectively performed a voxel-based analysis of the FA values in regions of hypoperfusion to identify a Tmax threshold at which ischemic changes can be identified. We found statistically significant higher FA values in the hypoperfused WM with Tmax≥5.4 s, suggesting that perhaps microstructural ischemic changes are largest above this threshold. Significant increase in FA values in the hypoperfused WM with Tmax ≥ 5.4 s supports the result of Diffusion and Perfusion Imaging Evaluation for Understanding Stroke Evolution (DEFUSE) trial,^12^ suggesting that Tmax>6 s may represent a threshold for differentiating ischemic penumbra from benign oligemia. Recently, the hypoperfusion intensity ratio, defined as the proportion of Tmax>6 s lesion volume with a Tmax>10-s delay has been proposed as a good predictive measure of infarction growth and clinical outcome.^26^ It will be interesting to compare the FA values against the hypoperfusion intensity ratio in the future and in a more broad clinical setting.

This study has several limitations, including (1) a relatively small sample size drawn from a single institution possibly introducing a sample bias; (2) the FA values likely resulted from a combination of ischemic injury and edema-induced compression and distortion of WM tracts, making it difficult to differentiate the 2 underlying pathophysiologies although within 6 hours of symptoms onset the effect of edema should be modest; (3) using FA>0.15 to generate the gray matter mask is suboptimal. Ideally, high-resolution T2- or T1-weighted imaging can be used for white-gray matter segmentation if available; (4) we used an ADC threshold of <600×10^−6^ mm^2^/s as a cutoff for infarction core as suggested by previous reports^3,27^ and, therefore, the distinction between ischemia and irreversible infarction should be interpreted in this context; and (5) the FA changes in infarction and ischemic region are variable depending on the severity of ischemia and time of onset. Our results represent the changes in patients within the first 6 hours and, therefore, the results should be interpreted in this context. It should be emphasized that detection of this acute elevation in FA is challenging because the increase is not large, has a short time course, and relies on comparison with a control regions of interest. It is, however, plausible that FA changes related to hyperacute ischemia could be used quantitatively for better differentiation of ischemic changes and infarction core in comparison with currently used diffusion-weighted imaging-ADC method alone and assessment of its potential clinical use would be an important next step for future studies.

## Conclusions

DTI-measured FA is decreased in regions of WM infarction and increased in hypoperfused, but not infarcted, WM in patients with hyperacute AIS. The FA values are significantly higher in the hypoperfused WM with Tmax≥5.4 s suggestive of early and perhaps real microstructural changes related to ischemia.

## Acknowledgments

We thank Jeff Gornbein/University of California, Los Angeles SBCC for statistical analysis support.

## Disclosures

Dr Nael is a consultant to Olea Medical; honorarium, unrelated to the subject of this project. The other authors report no conflicts.

## References

[1] Moseley ME, Kucharczyk J, Mintorovitch J, Cohen Y, Kurhanewicz J, Derugin N (1990). Diffusion-weighted MR imaging of acute stroke: correlation with T2-weighted and magnetic susceptibility-enhanced MR imaging in cats.. AJNR Am J Neuroradiol.

[2] Mintorovitch J, Yang GY, Shimizu H, Kucharczyk J, Chan PH, Weinstein PR (1994). Diffusion-weighted magnetic resonance imaging of acute focal cerebral ischemia: comparison of signal intensity with changes in brain water and Na+,K(+)-ATPase activity.. J Cereb Blood Flow Metab.

[3] Dardzinski BJ, Sotak CH, Fisher M, Hasegawa Y, Li L, Minematsu K (1993). Apparent diffusion coefficient mapping of experimental focal cerebral ischemia using diffusion-weighted echo-planar imaging.. Magn Reson Med.

[4] Sorensen AG, Copen WA, Ostergaard L, Buonanno FS, Gonzalez RG, Rordorf G (1999). Hyperacute stroke: simultaneous measurement of relative cerebral blood volume, relative cerebral blood flow, and mean tissue transit time.. Radiology.

[5] Rosen BR, Belliveau JW, Aronen HJ, Kennedy D, Buchbinder BR, Fischman A (1991). Susceptibility contrast imaging of cerebral blood volume: Human experience.. Magn Reson Med.

[6] Schlaug G, Benfield A, Baird AE, Siewert B, Lövblad KO, Parker RA (1999). The ischemic penumbra: operationally defined by diffusion and perfusion MRI.. Neurology.

[7] Neumann-Haefelin T, Wittsack HJ, Wenserski F, Siebler M, Seitz RJ, Mödder U (1999). Diffusion- and perfusion-weighted MRI. The DWI/PWI mismatch region in acute stroke.. Stroke.

[8] Hacke W, Furlan AJ, Al-Rawi Y, Davalos A, Fiebach JB, Gruber F (2009). Intravenous desmoteplase in patients with acute ischaemic stroke selected by MRI perfusion-diffusion weighted imaging or perfusion CT (DIAS-2): a prospective, randomised, double-blind, placebo-controlled study.. Lancet Neurol.

[9] Kidwell CS, Jahan R, Gornbein J, Alger JR, Nenov V, Ajani Z, MR RESCUE Investigators (2013). A trial of imaging selection and endovascular treatment for ischemic stroke.. N Engl J Med.

[10] Thijs VN, Somford DM, Bammer R, Robberecht W, Moseley ME, Albers GW (2004). Influence of arterial input function on hypoperfusion volumes measured with perfusion-weighted imaging.. Stroke.

[11] Shih LC, Saver JL, Alger JR, Starkman S, Leary MC, Vinuela F (2003). Perfusion-weighted magnetic resonance imaging thresholds identifying core, irreversibly infarcted tissue.. Stroke.

[12] Wheeler HM, Mlynash M, Inoue M, Tipirneni A, Liggins J, Zaharchuk G, DEFUSE 2 Investigators (2013). Early diffusion-weighted imaging and perfusion-weighted imaging lesion volumes forecast final infarct size in DEFUSE 2.. Stroke.

[13] Bhagat YA, Hussain MS, Stobbe RW, Butcher KS, Emery DJ, Shuaib A (2008). Elevations of diffusion anisotropy are associated with hyper-acute stroke: a serial imaging study.. Magn Reson Imaging.

[14] Carano RA, Li F, Irie K, Helmer KG, Silva MD, Fisher M (2000). Multispectral analysis of the temporal evolution of cerebral ischemia in the rat brain.. J Magn Reson Imaging.

[15] Ozsunar Y, Grant PE, Huisman TA, Schaefer PW, Wu O, Sorensen AG (2004). Evolution of water diffusion and anisotropy in hyperacute stroke: significant correlation between fractional anisotropy and T2.. AJNR Am J Neuroradiol.

[16] Griswold MA, Jakob PM, Heidemann RM, Nittka M, Jellus V, Wang J (2002). Generalized autocalibrating partially parallel acquisitions (GRAPPA).. Magn Reson Med.

[17] Wu O, Østergaard L, Weisskoff RM, Benner T, Rosen BR, Sorensen AG (2003). Tracer arrival timing-insensitive technique for estimating flow in MR perfusion-weighted imaging using singular value decomposition with a block-circulant deconvolution matrix.. Magn Reson Med.

[18] Basser PJ, Pierpaoli C (1998). A simplified method to measure the diffusion tensor from seven MR images.. Magn Reson Med.

[19] Green HA, Peña A, Price CJ, Warburton EA, Pickard JD, Carpenter TA (2002). Increased anisotropy in acute stroke: a possible explanation.. Stroke.

[20] Yang Q, Tress BM, Barber PA, Desmond PM, Darby DG, Gerraty RP (1999). Serial study of apparent diffusion coefficient and anisotropy in patients with acute stroke.. Stroke.

[21] Zelaya F, Flood N, Chalk JB, Wang D, Doddrell DM, Strugnell W (1999). An evaluation of the time dependence of the anisotropy of the water diffusion tensor in acute human ischemia.. Magn Reson Imaging.

[22] Albers GW, Thijs VN, Wechsler L, Kemp S, Schlaug G, Skalabrin E, DEFUSE Investigators (2006). Magnetic resonance imaging profiles predict clinical response to early reperfusion: the diffusion and perfusion imaging evaluation for understanding stroke evolution (DEFUSE) study.. Ann Neurol.

[23] Davis SM, Donnan GA, Parsons MW, Levi C, Butcher KS, Peeters A, EPITHET Investigators (2008). Effects of alteplase beyond 3 h after stroke in the Echoplanar Imaging Thrombolytic Evaluation Trial (EPITHET): a placebo-controlled randomised trial.. Lancet Neurol.

[24] Lansberg MG, Straka M, Kemp S, Mlynash M, Wechsler LR, Jovin TG, DEFUSE 2 Study Investigators (2012). MRI profile and response to endovascular reperfusion after stroke (DEFUSE 2): a prospective cohort study.. Lancet Neurol.

[25] Zaharchuk G (2007). Theoretical basis of hemodynamic MR imaging techniques to measure cerebral blood volume, cerebral blood flow, and permeability.. AJNR Am J Neuroradiol.

[26] Olivot JM, Mlynash M, Inoue M, Marks MP, Wheeler HM, Kemp S, DEFUSE 2 Investigators (2014). Hypoperfusion intensity ratio predicts infarct progression and functional outcome in the DEFUSE 2 Cohort.. Stroke.

[27] Purushotham A, Campbell BC, Straka M, Mlynash M, Olivot JM, Bammer R Apparent diffusion coefficient threshold for delineation of ischemic core [published online ahead of print June 27, 2013].. Int J Stroke.

